# The Diagnostic Trap Occurred in Two COVID-19 Cases Combined Pneumocystis Pneumonia in Patient with AIDS

**DOI:** 10.21203/rs.3.rs-53350/v1

**Published:** 2020-08-10

**Authors:** Wei Guo, Maomao Wang, Fangzhao Ming, Weiming Tang, Ke Liang

**Affiliations:** Zhongnan Hospital of Wuhan University; Changhai Hospital of the Second Military Medical University; Wuchang District Center for Disease Control and Prevention; Southern Medical University, and the University of North Carolina at Chapel Hill Project-China; Zhongnan Hospital of Wuhan University

**Keywords:** diagnostic trap, HIV, SARS-CoV-2, Co-infection, COVID-19, Pneumocystis Pneumonia

## Abstract

**Background:**

The limited knowledge on the diagnosis of Coronavirus disease 2019 (COVID-19) at the early stage of the pandemic may lead to misdiagnoses, especially when the nucleic acid inspection cannot meet the mass requirement. This condition is even actual for people who are living with HIV/AIDS (PLWHA), for the latter is vulnerable to variable infections.

**Case Presentation:**

In this short communication, we introduced two HIV infected individuals who had PCP but was misdiagnosed as COVID-19 initially, and finally infected with SARS-CoV-2 in hospital in Wuhan, China. Eventually, both patients improved soon after they were switched to the treatment of SMZ/TMP.

**Conclusions:**

We suggested that the hospitalized COVID-19 cases should be screened with HIV and other pathogens, to prevent that PLWHA who have PCP from being misdiagnosed as COVID-19.

## Background

The limited knowledge on the diagnosis of Coronavirus disease 2019 (COVID-19) at the early stage of the pandemic may lead to the misdiagnosis of the diseases. This condition is even actual for people who are living with HIV/AIDS (PLWHA). In the recent case published in the International Journal of Infectious Diseases, we reported a case about an AIDS patient co-infected with SARS-CoV-2 in Wuhan, China^1^. However, after following up and reviewing the timeline of the patient's clinical process, we found that this patient was suffered from Pneumocystis Pneumonia (PCP) initially, then he infected SARS-CoV-2 in hospital. In this short communication, we introduced two HIV infected individuals, including the reported one (designated as P1 in this report), who had PCP and misdiagnosed as COVID-19, and finally infected with SARS-CoV-2 in hospital in Wuhan, China.

## Case Presentation

In middle February, to move suspected cases in Hubei province to care, the diagnosis criteria of COVID-19 changed to 1) living in the epidemic center, 2) presented with typical symptoms of COVID-19, 3) the radiographic presentation showed ground-glass opacities (GGO) and interstitial pneumonia, no matter the SARS-CoV-2 nucleic acid test (NAT) is negative or positive^2^. This change quickly moved many suspected cases to treatment but increased the opportunity of misdiagnoses^3^. The two cases discussed in this commentary piece are examples of the misdiagnosis. These two cases presented high fever and dyspnea on admission to hospitals in February 2020 (35 days and 11 days from the onset of the symptoms to the date of admission), both had GGO and interstitial pneumonia in chest CT, and were NAT negative for SARS-CoV-2 before admission (Table).

After charged into isolated wards and treated with anti-viral medications plus corticosteroids, patient one (P1) progressively deteriorated in the symptoms, while patient two (P2) retained the symptoms during this treatment. For P1, he concealed that he was living with HIV, and the NAT testing turned positive on day nine after admission. The specific antibodies for SARS-CoV-2 were negative on day 24 of admission and turned to IgM positive on day 28 (Figure). After acquired his HIV positive result, he was considered as PCP and was treated with trimethoprim-sulfamethoxazole (TMP/SMZ) and then switched to clindamycin because the patient was allergic to TMP/SMZ on day 31, and the status of the patient was improved soon. The patient was considered to be infected with SARS-CoV-2 in the isolation wards and finally turned positive after the admission. To be noted, on the 65th day after he was tested positive of NAT, both IgM and IgG turned negative, which indicated that effective immune defense against SARS-CoV-2 was hard to build in PLWHA.

As for P2, the NATs were negative throughout the whole course of the disease, while specific IgM for SARS-CoV-2 was positive on day 18 (which was not tested before), and IgG was positive after that (day 27). After being diagnosed as HIV positive on day 20, the patient was considered and treated as PCP (TMP/SMZ). His status was also improved soon after treatment for PCP. In summary, the chronic onset of his clinical course, high lactate dehydrogenase (LDH), interstitial pneumonia by CT scan, and effective specific treatment for PCP favored the diagnosis for PCP from the onset of the disease. For P2, we can not exclude the possibility that the patient was infected with SARS-CoV-2 in the isolation ward.

## Discussion And Conclusions

Along with knowledge of COVID-19's clinical features and the progress of detection methods, people began to aware of the misdiagnosis of PCP as COVID-19^4^. We have also found similar cases in the clinic. This commentary piece reported two AIDS/ PCP patients who were misdiagnosed as COVID-19 initially, which led to their nosocomial infection of SARS-COV-2, providing a useful reference for clinical practice. In the epidemic region of COVID-19, for PLWHA with low CD4 count who presented GGO and interstitial pneumonia in CT scan, PCP should be differentiated with COVID-19. Although nearly all viral pneumonia and a few other cases of pneumonia presented with GGO and interstitial pneumonia in CT, and both cases had no pathogen evidence, the long term onset of clinical symptoms, low CD4 count, and the quick response to TMP/SMZ treatment helped us to diagnose PCP.

Combined bacterial infection with COVID-19 is correlated with higher mortality^5^. PCP is demonstrated as a dangerous opportunistic infection in PLWHAs^6^. However, in this two PCP co-infected with COVID-19 patients, although the PaO_2_ of P2 was low, high flow oxygen plus anti-PCP treatment cured the patients. Eventually, both patients presented with a significantly improved radiograph, none symptoms, and negative NAT by the time they were discharged from hospitals.

This clinic experience demonstrated that the hospitalized COVID-19 cases should be screened with HIV and other pathogens, to prevent that PLWHA who have PCP from being misdiagnosed as COVID-19^7^, of which may not only lead to hospital-acquired infection of SARS-CoV-2 but also missed the most important opportunity in providing timely care to patients with PCP.

## Figures and Tables

**Figure 1:**
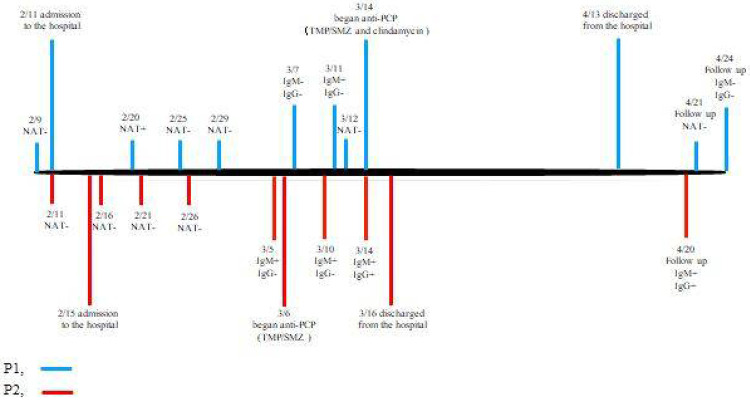
The timeline for the clinical causes of Patient 1 (on the upper panel) and patient 2 (on the lower panel). PCP: Pneumocystis pneumonia, NAT: nucleic acid test, TMP/SMZ: trimethoprim-sulfamethoxazole.

## References

[1] Wang M, Luo L, Bu H, Xia H. One case of coronavirus disease 2019 (COVID-19) in a patient co-infected by HIV with a low CD4(+) T-cell count. International journal of infectious diseases: IJID : official publication of the International Society for Infectious Diseases. 2020; 96:148–50. 10.1093/cid/ciaa423 32335339 PMC7194654

[2] Commission CNH. Diagnosis and treatment of 2019-nCoV pneumonia in China (the 5th edition). *In* Chinese *Published* 2 4, 2020 Accessed February 8, 2020 http://www.nhc.govcn/yzygj/s7653p/202002/d4b895337e19445f8d728fcaf1e3e13ashtml 2020.

[3] Weiming T, Huipeng L, Gifty M, The changing pattern of COVID-19 in China: A tempogeographic analysis of the SARS-CoV-2 epidemic. Clin Infect Dis. 2020. doi:10.1093/cid/ciaa423 PMC7184457 32296826

[4] Choy CY, Wong CS. It's not all about COVID-19: pneumocystis pneumonia in the era of a respiratory outbreak. J Int AIDS Soc 2020; (23). DOI:10.1002/jia2.25533 PMC7273113 32558276

[5] Cox MJ, Loman N, Bogaert D, O'Grady J. Co-infections: potentially lethal and unexplored in COVID-19. The Lancet Microbe 2020; 1(1). Doi:10.1016/s2666-5247(20)30009-4 PMC7195315 32835323

[6] Adolescents. PoOIiH-IAa. Guidelines for the prevention and treatment of opportunistic infections in HIV-infected adults and adolescents: recommendations from the Centers for Disease Control and Prevention, the National Institutes of Health, and the HIV Medicine Association of the Infectious Diseases Society of America. Available at http://aidsinfo.nih.gov/contentfiles/lvguidelines/adult_oi.pdf. Accessed May 14th, 2020.

[7] Tian Xinlun P, Min W, Hanping, The differential diagnosis for novel coronavirus pneumonia and similar lung diseases in general hospitals. Zhonghua jie he he hu xi za zhi = Zhonghua jiehe he huxi zazhi = Chinese journal of tuberculosis and respiratory diseases 2020 Doi: 10.3760/cma.j.cn112147-20200221-00136 32153167

